# Die 2024 ESC-Leitlinien zum Management von Vorhofflimmern: AF-CARE als neues Credo

**DOI:** 10.1007/s00399-024-01053-7

**Published:** 2024-11-13

**Authors:** Julian Wolfes, Lars Eckardt

**Affiliations:** Klinik für Kardiologie II – Rhythmologie, Universitätsklinikum Münster, Albert-Schweitzer Campus 1, 48149 Münster, Deutschland

**Keywords:** Vorhofflimmern, ESC Leitlinie, Evidenz-basierte Therapie, Update, Vorhofflimmermanagement, Atrial fibrillation, ESC guidelines, evidence-based treatment, Update, AF management

## Abstract

Die neue ESC-Leitlinie zum Management von Patienten mit Vorhofflimmern von 2024 führt den *AF-CARE-*Pfad als zentrales Akronym des Vorhofflimmermanagements ein. In diesem Zuge rückt das Management von Komorbiditäten (*Comorbidities*) und Risikofaktoren an die erste Stelle des Vorhofflimmermanagements. Aber auch bei der Schlaganfall- und Thromboembolie-Prophylaxe (*Avoidance*) zeigt die neue Leitlinie wichtige Änderungen, wie einen veränderten Risikoscore (CHA_2_DS_2_-VA) sowie eine Stellungnahme zur Antikoagulation bei subklinischem Vorhofflimmern. Ebenfalls finden sich Änderungen in den Konzepten der Rhythmus- und Frequenzkontrolle mit einer Aufwertung der Rhythmuskontrolle und der Katheterablation. Schlussendlich empfiehlt die Leitlinie eine regelmäßige Reevaluation des Patientenverlaufs zum optimalen Vorhofflimmermanagement. Diese Übersicht fasst die wesentlichen Neuerungen zusammen und diskutiert einige Empfehlungen zu Aspekten, die auch anders bewertet werden können.

## Abstract (en)

The new 2024 ESC guidelines on the management of patients with atrial fibrillation (AF) introduces the *AF-CARE* pathway as the central acronym for AF management. As a result, the management of Comorbidities and risk factors is moving to the forefront of AF management. However, the new guideline also includes important changes in Avoidance stroke and thromboembolism, such as a modified risk score (CHA_2_DS_2_-VA) and a statement on anticoagulation in subclinical atrial fibrillation. There are also changes in the concepts of Rhythm and rate control with an upgrading of rhythm control and catheter ablation. Finally, the guideline recommends regular Evaluation and reassessment of the patient’s course for optimal AF management. Here, we summarize key points of the new guidelines and discusses some recommendations on aspects that can also be assessed differently.

## Einleitung

Die neuen europäischen Vorhofflimmer-Leitlinien, die von der Europäischen Gesellschaft für Kardiologie auf ihrer Jahrestagung im September 2024 veröffentlicht wurden, sollen eine zeitgemäße und evidenzbasierte Diagnostik und Behandlung von Vorhofflimmern sicherstellen. Im Folgenden werden die wichtigsten Implikationen der neuen Leitlinie für die kardiologische Versorgungspraxis diskutiert.

### Grundlegendes neues Akronym

**Abb. 1:**
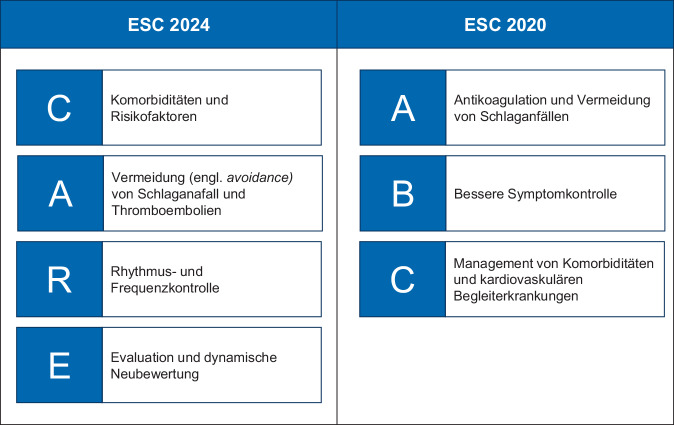
Akronyme des Vorhofflimmermanagements im Vergleich zwischen der ESC-Leitlinie 2020 und 2024

Die zentrale Neuerung der Leitlinie ist das sog. *AF-CARE-Konzept*, das mit nahezu 100 Nennungen die Leitlinie als Credo wie eine neue Maxime durchzieht und dessen Anwendung im Alltag auf Basis der Leitlinien Task Force eine Klasse-IC-Empfehlung erhalten hat. Es folgt auf das ABC-Konzept [1] der bisherigen Leitlinie (Abb. 1). AF-CARE betont die zentralen Behandlungsaspekte: Diagnose und Therapie von Komorbiditäten und Risikofaktoren (*C*), Vermeidung von Thromboembolien (*A*), Frequenz- und Rhythmuskontrolle (*R*) sowie Evaluation und kontinuierliche Nachsorgen (*E*). Die Abfolge der einzelnen Punkte unterstreicht die nach Ansicht der Autoren zentrale Bedeutung des Managements von Komorbiditäten und Risikofaktoren in der Versorgung von Patienten mit Vorhofflimmern. Diese Einschätzung betont zu Recht die Bedeutung präventiver Maßnahmen in der Behandlung von Vorhofflimmern, lässt aber anderseits außer Acht, dass unverändert im klinischen Alltag insbesondere beim Erstkontakt mit einem Vorhofflimmerpatienten die Risikoeinstufung bzgl. thromboembolischer Ereignisse und ggf. der unmittelbare Beginn einer Antikoagulation im Vordergrund stehen sollten. Vordergründig lässt sich diese Reihenfolge in AF-CARE mit *Vorhofflimmer-Pflege* oder -*Sorgfalt* sprachlich elegant und inhaltlich umsorgend abbilden. AF-ACRE oder AF-AREC klingt so hölzern, dass man auch bei ABC hätten bleiben können; und dies vielleicht auch aus guten Gründen: In vierjährigen Abständen handlungsweisende Akronyme zu ändern, erleichtert die Umsetzung von Leitlinien in die klinische Praxis sicherlich nicht.

Trotz aller Innovationen im Bereich der künstlichen Intelligenz [2], aber vor allem auch neuer Screeningverfahren (u. a. Wearables wie Smart-Watches), erfordert die Diagnose von Vorhofflimmern nach wie vor die Dokumentation mittels 1‑Kanal- oder Mehrkanal-EKG. Die regelmäßige Aufzeichnung des Herzrhythmus für das Vorhofflimmerscreening wird für Personen ≥ 65 Jahren im Zuge regulärer Arztkontakte empfohlen (opportunistisches Screening). Ein längeres EKG-basiertes „population-based“ Screening (systematisches Screening) sollte auch für Personen im Alter von > 75 Jahren oder > 65 Jahren mit thromboembolischen Risikofaktoren erwogen werden [3], obwohl bislang nicht gezeigt werden konnte, dass durch ein Vorhofflimmerscreening das Risiko für die Entwicklung von fortschreitenden Rhythmusstörungen oder Begleitkomplikationen [4] signifikant gesenkt werden kann und aktuelle Studien wie STROKESTOP II zudem die geringe allgemeine Breitschaft, am Screening teilzunehmen, unterstreichen [5].

Die Leitlinie erkennt auch die technologische Innovation des Photoplethysmographie- und KI-basierten Vorhofflimmerscreenings an, obwohl diese (bislang) nicht als ausreichend für die Diagnose von Vorhofflimmern angesehen werden. Neu in der Leitlinie ist die explizite Forderung, dass nach der Erstdiagnose von Vorhofflimmern eine transthorakale Echokardiographie erfolgen sollte, um weitere Behandlungsentscheidungen einzuleiten. Bemerkenswerterweise werden pathophysiologische Aspekte wie die Rolle einer atrialen Kardiomyopathie und/oder die Bedeutung atrialer Fibrose kaum adressiert. So spielt auch die kardiale Magnetresonanztomographie (MRT) in der Leitlinie keine Rolle, was angesichts methodischer Limitationen der MRT auf Vorhofebene, aber auch klinischen Befunden wie DECAAF II [6] nachvollziehbar ist, aber auch hätte kommentiert werden können. Dies wurde aktuell in einem Konsensuspapier von EHRA und ESC zur atrialen Kardiomyopathie adressiert [7].

## Individualisierte Therapie von Komorbiditäten und Risikofaktoren

**Abb. 2:**
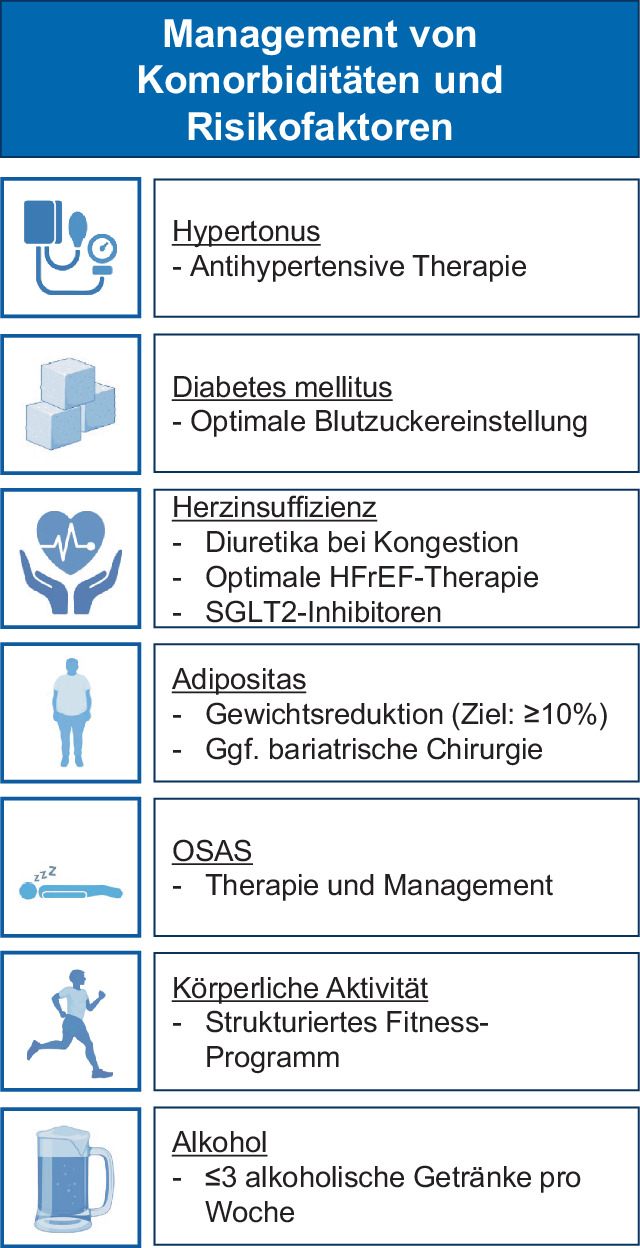
Wichtige Kernbotschaften und Änderungen zum Management von Komorbiditäten und Risikofaktoren

Die individualisierte Therapie von Komorbiditäten und Risikofaktoren ist zentraler Bestandteil der neuen Leitlinie (Abb. 2). Gegenüber 2020 wurden zahlreiche Aspekte von Klasse-IIa- zu Klasse-I-Empfehlungen heraufgestuft (u. a. Gewichtsreduktion, Alkoholreduktion, gezieltes körperliches Training). Diese ergänzen eine Reihe weiterer Klasse-I-Empfehlungen zur Hypertonie- und Herzinsuffizienzbehandlung sowie zur Einstellung von Diabetes. Bei Herzinsuffizienz empfiehlt die Leitlinie in Analogie zu anderen ESC-Leitlinien SGLT2-Inhibitoren für Patienten mit Vorhofflimmern und Herzinsuffizienz, unabhängig von der LVEF [8]. Bei Patienten mit Diabetes mellitus oder Prädiabetes wird eine optimale Blutzuckerkontrolle empfohlen, wobei Metformin und SGLT2-Iinhibitoren eine besondere Rolle in der Prophylaxe von Vorhofflimmern bei Typ-II-Diabetikern zugeschrieben wird. In der aktuellen Leitlinie wird Adipositas als wichtige Komorbidität von Vorhofflimmern diskutiert. Im Gegensatz zur vorherigen Leitlinie wird explizit eine Gewichtsreduktion von ≥ 10 % des Körpergewichts empfohlen [9]. Zahlreiche Studien belegen erst ab dieser Größenordnung einen positiven Effekt auf Vorhofflimmern. In Einzelfällen kann auch eine bariatrische Operation zur Reduzierung der Vorhofflimmerbelastung in Betracht gezogen werden [10]. Wichtig erscheint auch die Behandlung schlafbezogener Atmungsstörungen, wobei die neue Leitlinie in der Diagnostik der obstruktiven Schlafapnoe (OSAS) von symptombasierten Fragebögen abrät, da Polysomnographie oder Heim-Schlafapnoe-Tests weitaus zuverlässiger sind [11]. Die Leitlinie enthält ebenfalls klare Empfehlungen zu körperlicher Aktivität. Zur Vorbeugung von Vorhofflimmern werden 150 bis 300 min moderate körperliche Aktivität pro Woche oder 75 bis 150 min intensive aerobe körperliche Aktivität pro Woche empfohlen. Der Alkoholkonsum sollte auf maximal 30 g Alkohol bzw. 3 alkoholische Getränke pro Woche reduziert werden [12].

## Antikoagulation und Schlaganfallprophylaxe

**Abb. 3:**
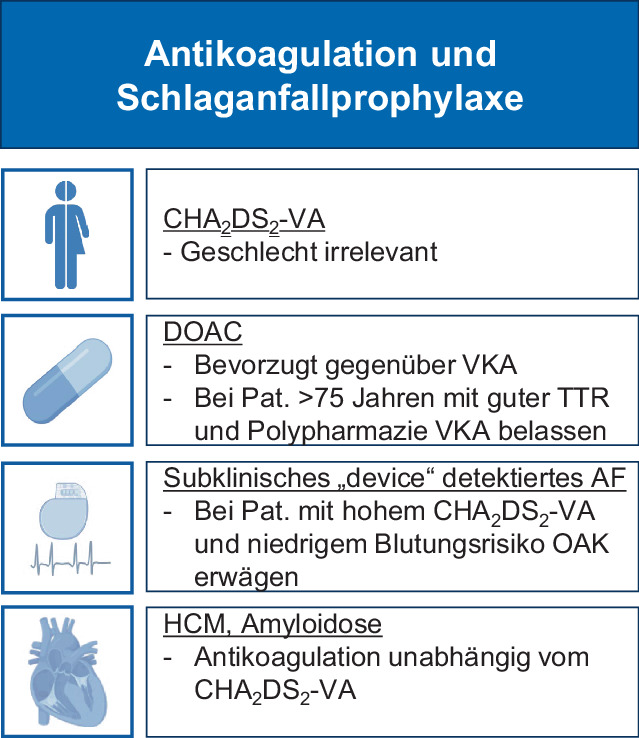
Wichtige Kernbotschaften und Änderungen zur Antikoagulation und Schlaganfallprophylaxe

Die ESC-Leitlinie empfiehlt eine Antikoagulation primär mit direkten oralen Antikoagulanzien (DOAC) bei Patienten mit Vorhofflimmern und Risikofaktoren für das Auftreten eines thromboembolischen Ereignisses (Abb. 3). Neu ist hier die Verwendung des CHA_2_DS_2_-VA-Scores als Modifikation zum bisher verwendeten CHA_2_DS_2_-VASc-Score, so dass das Geschlecht nicht mehr in den Score eingeht und die Empfehlungen geschlechtsunabhängig vereinheitlicht werden. Dies Entwicklung ist zeitgemäß, seit 2016 wurde in den ESC-Leitlinien auf die geringe Bedeutung des weiblichen Geschlechts als Risikofaktor hingewiesen. Aktuelle Daten unterstützen diese Entscheidung [13]. Ein CHA_2_DS_2_-VA-Score von mehr als einem Punkt stellt demnach jetzt geschlechtsunabhängig eine Klasse-I-Empfehlung für eine orale Antikoagulation (OAK) dar. Ein CHA_2_DS_2_-VA-Score von einem Punkt ist unverändert eine Klasse-IIa-Empfehlung zur OAK. Bei Patienten, die ein subklinisches Vorhofflimmern in Form atrialer Hochfrequenzepisoden in kardialen Devices („device-detected AF“) aufweisen [14], ist die Leitlinie, aufgrund der Daten aus der NOAH-AFNET 6 [15] und der ARTESiA-Studie [16] zurückhaltend mit einer Antikoagulationsempfehlung. Dies ist nachvollziehbar, wenn man berücksichtigt, dass sich aus der ARTESiA-Studie, die randomisiert Apixaban versus ASS verglichen hat, ein sehr geringer Nutzen für die OAK bei vermehrten Blutungen gezeigt hat. So errechnet sich aus ARTESiA eine NNT zur Verhinderung eines klinisch bedeutsamen Schlaganfalls (Rankin-Score 3–6) von über 350 [14]. Die Leitlinie empfiehlt lediglich für Subgruppen mit hohem Schlaganfall- und geringem Blutungsrisiko eine Antikoagulation im Einzelfall zu erwägen (Klasse-IIb-Indikation). Vor dem Hintergrund der Daten zu subklinischem Vorhofflimmern hätte man sich eine detailliertere Diskussion zur Bedeutung der Vorhofflimmerlast („AF burden“) und auch der Frage, ob die historischen 30 s einer EKG-Aufzeichnung für die Diagnose von Vorhofflimmern noch zeitgemäß sind, gewünscht.

Tritt während einer bestehenden Antikoagulation ein Schlaganfall auf, empfiehlt die aktuelle Vorhofflimmer-Leitlinie keinen standardmäßigen Wechsel des eingenommenen DOAC oder die Ergänzung der bestehenden Antikoagulation durch eine Thrombozytenaggregationsbehandlung [17] (Klasse-III-Empfehlung). Explizit wird im Übrigen betont, dass eine Dosisreduktion eines DOAC nur bei entsprechender Indikation gemäß den jeweiligen Fachinformationen erfolgen sollte (Klasse-III-Empfehlung).

Für Patienten, die sich einer Herzoperation mit bekanntem Vorhofflimmern unterziehen, empfiehlt die neue Leitlinie auf Grundlage der Daten aus der LAAOS-III-Studie einen LAA-Verschluss während der Operation, um thromboembolische Komplikationen zu verringern [18]. Die Antikoagulation sollte allerdings (wie in der zugrundeliegenden Studie) fortgesetzt werden. Die aktuelle Leitlinie empfiehlt auch eine langfristige wirksame Antikoagulation bei postoperativem Vorhofflimmern und thromboembolischen Risikofaktoren (Klasse IIa) oder bei Trigger-induziertem (z. B. Sepsis, Perikarditis, OSAS) Vorhofflimmern (Klasse I). Hier zeigt sich ein Grundtenor der Leitlinie „safety first“ mit einem bestmöglichen Schutz vor thromboembolischen Ereignissen, auch wenn die Datenlage für die genannten Aspekte alleinig auf Beobachtungsstudien beruht. Ebenso wird eine Antikoagulation unabhängig vom CHA_2_DS_2_-VA-Score bei Vorhofflimmerpatienten mit angeborenem Herzfehler und chirurgischer intrakardialer Reparatur, Zyanose, Fontan-Palliation oder systemischem rechtem Ventrikel empfohlen.

## Frequenz- und Rhythmuskontrolle

Grundsätzlich wird in der aktuellen Leitlinie auf der Grundlage der der RACE-II-Studie [19] unverändert insbesondere beim Erstkontakt und tachykardem Vorhofflimmern eine Frequenzkontrolle mit einer Herzfrequenz < 110/min empfohlen. Zur Frequenzkontrolle werden Betablocker und Digoxin unabhängig von der LVEF und zusätzlich Diltiazem/Verapamil für Patienten mit einer LVEF > 40 % empfohlen. Hier haben die Daten der RATE-AF-Studie [20] zu einer Aufwertung des Empfehlungsgrades von Digoxin geführt. In RATE-AF wurden Patienten mit permanentem Vorhofflimmern auf eine Bisoprolol- oder Digoxin-Therapie randomisiert und als primärer Endpunkt die Lebensqualität untersucht, wobei sich ein geringer Vorteil zugunsten von Digoxin zeigte.

**Abb. 4:**
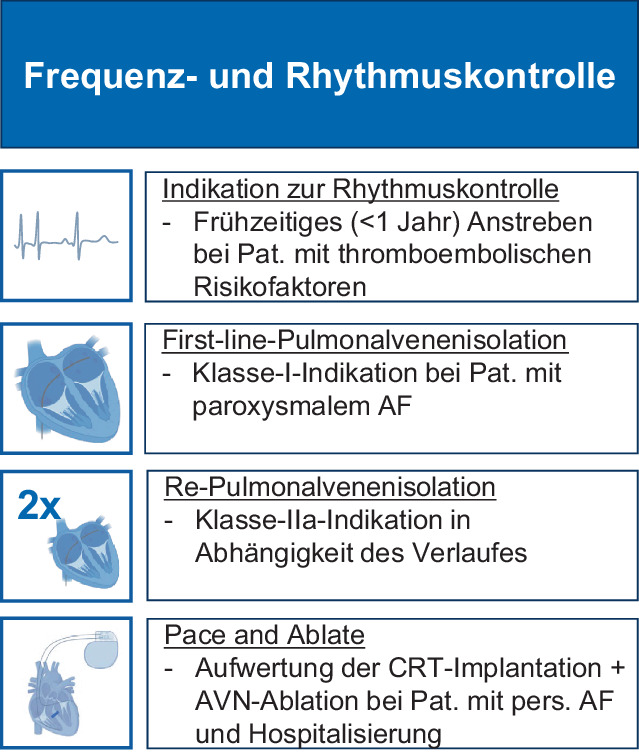
Wichtige Kernbotschaften und Änderungen zur Frequenz- und Rhythmuskontrolle

Die Möglichkeit der Schrittmacherimplantation mit sekundärer AV-Knotenablation („Pace-and-ablate-Strategie“) oder AV-Knotenablation bei Patienten mit implantiertem Schrittmacher hat in der aktuellen Leitlinie eine IIa-Indikation für Patienten erhalten, die für eine pharmakologische Frequenzkontrolle nicht geeignet sind (Abb. 4). Die Daten der APAF-CRT-Studie [21], die eine CRT-Implantation mit AV-Knoten-Ablation randomisiert mit einer pharmakologische Frequenzkontrolle bei Patienten mit symptomatischem persistierendem Vorhofflimmern und mindestens einem vorherigen herzinsuffizienzbedingten Krankenhausaufenthalt verglichen hat, wurden aufgenommen. Aufgrund des signifikanten Vorteils im CRT-Arm im Hinblick auf den primären Endpunkt der Studie (Tod oder Hospitalisierung bei Herzinsuffizienz) sieht die Leitlinie in diesen Fällen eine IIa-Indikation für die CRT-Implantation mit AV-Knoten-Ablation vor. Aktuelle Studien untersuchen darüber hinaus den Stellenwert einer Pace-and-ablate-Strategie bei älteren Patienten [22], denn gerade für diese zeigen Registerdaten aus Deutschland eine signifikanten Nutzen dieser Strategie im Hinblick auf die Lebensqualität [23]. Bei neu aufgetretenem Vorhofflimmern und hämodynamischer Stabilität sollte basierend auf den Daten der RACE 7-ACWAS-Studie [24] eine Watch-and-wait-Strategie erwogen werden. Aufgrund der sehr hohen Spontankonversionsraten können im ambulanten Bereich und in den Notaufnahmen so Ressourcen gespart werden (in RACE 7 terminierte bei 69 % der Patienten das Vorhofflimmern innerhalb von 48 h).

### Rhythmuskontrolle – erstmals Berücksichtigung einer prognostischen Indikation

In der neuen Leitlinie wird betont, dass die Rhythmuskontrolle keine eigenständige Behandlung darstellt, sondern immer in den AF-CARE-Pfad integriert werden sollte. Neu ist, dass bei jedem Patienten die Option einer Rhythmuskontrolle betrachtet und damit geprüft werden sollte. Die Indikation zur Rhythmuskontrolle wurde bislang überwiegend basierend auf mit Vorhofflimmern assoziierten Symptomen gestellt. Hier hat ein (bislang) vorsichtiges Umdenken in der neuen Leitlinie stattgefunden. Vor allem Ergebnisse der EAST-AFNET 4-Studie [25] stellen diese Grundannahme in Frage [26]. In der EAST-Studie wurden Patienten im ersten Jahr nach der Diagnose von Vorhofflimmern, die älter als 75 Jahre waren oder kardiovaskuläre Risikofaktoren aufwiesen, zu einer frühen Rhythmuskontrolle oder einer *konventionellen* Frequenzkontrolle randomisiert. Es zeigte sich eine Verringerung des kombinierten Endpunkts von kardiovaskulärem Tod, Schlaganfall, Hospitalisierung bei Herzinsuffizienz oder akutem Koronarsyndrom, so dass die Leitlinie erstmals aus prognostischer Sicht eine Rhythmuskontrolle für diese Patienten formuliert (Klasse IIa). Dabei ist zu beachten, dass die Mehrzahl der EAST-Patienten eine medikamentöse Rhythmuskontrolle erhielt und zumindest Subanalysen aus EAST-AFNET 4 nicht nachweisen konnten, dass eine Ablationsbehandlung (25 % der Patienten wurden im Rhythmusarm in EAST abladiert) entscheidend das Outcome beeinflusst hat [26].

### Katheterablation – Aufwertung der Indikation

Immer mehr Daten [27] sprechen für eine Erstlinien-Katheterablation bei Patienten mit Vorhofflimmern, was zu einer Klasse-I-Indikation für Patienten mit paroxysmalem Vorhofflimmern geführt hat. Bei Patienten mit persistierendem Vorhofflimmern ist die Evidenzlage eingeschränkter, wobei die strenge, historische Differenzierung zwischen paroxysmalem und persistierendem Vorhofflimmern, wie sie die Leitlinie fortführt, den klinischen Alltag unzureichend abbildet. Hier ist die US-amerikanische Leitlinie aus dem vergangenen Jahr [28], die Vorhofflimmern in Stadien mit kontinuierlichem Übergang einteilt, deutlich progressiver. Sehr viele Patienten mit formal persistierendem Vorhofflimmern haben kein nachweisbares endokardiales Substrat und profitieren von einer Pulmonalvenenisolation (PVI) genauso wie Patienten mit paroxysmalem Vorhofflimmern [29]. Deshalb erscheint die Klasse-IIb-Empfehlung der ESC-Leitlinie für eine Erstlinientherapie bei persistierendem Vorhofflimmern (zu) konservativ. Aus klinischer Sicht wäre eine Einteilung in nichtpermanentes und permanentes Vorhofflimmern zeitgemäßer gewesen. Unverändert zu der Leitlinie aus 2020 besteht aber für paroxysmales wie persistierendes AF nach erfolgloser medikamentöser antiarrhythmischer Therapie eine Klasse-I-Indikation zur Katheterablation. Wenig Beachtung findet hier das Risiko einer Progression des Vorhofflimmerns zu persistierenden Formen unter einer ineffektiven medikamentösen antiarrhythmischen Therapie sowie die schlechteren Aussichten auf eine dauerhafte Rhythmisierung bei einer späten Ablation [30, 31]. Ebenso besteht bei Patienten mit HFrEF und Vorhofflimmern mit Verdacht auf eine Tachymyopathie eine Klasse-I-Indikation für eine Katheterablation [32]. Unter anderem basierend auf der CASTLE-HTx-Studie [33] sollte eine Ablation individuell bei progredient herzinsuffizienten Patienten auch aus prognostischen Gründen erfolgen (Klasse-IIa-Empfehlung). Dass die Ergebnisse von CASTLE-HTx nicht zu einer Klasse-I-Empfehlung geführt haben, mag man kritisieren, ist aber u. a. angesichts des Studiendesigns einer randomisierten nichtverblindeten Singlecenterstudie und der Größe der Studie sowie weiterer methodischer Limitationen nachvollziehbar [34].

Eine vollständige elektrische Isolation der Pulmonalvenen ist nach wie vor Ziel der Katheterablation von Vorhofflimmern; der Nutzen weiterer Ablationsläsionen ist bislang kaum bis nicht belegt und wird deshalb nicht generell empfohlen. Im Falle eines Rezidivs nach erfolgreicher Ablation sollte eine erneute PVI in Betracht gezogen werden. Im Einzelfall können auch endoskopische oder hybride Ablationsstrategien in erfahrenen Zentren zur effektiveren Kontrolle des Vorhofflimmerns gewählt werden [35]. Insgesamt wurde die chirurgische Vorhofflimmertherapie aufgewertet, so besteht eine Klasse-I-Empfehlung für Patienten mit symptomatischem Vorhofflimmern und chirurgischer Mitralklappen Therapie. Inwieweit der Einsatz neuer Energieformen wie die Pulsed-field-Ablation [36] solche hybriden Ablationskonzepte flankieren oder ersetzen können, ist derzeit noch Gegenstand der Forschung.

In Abhängigkeit vom CHA_2_DS_2_-VA-Score sollte mindestens 3 Wochen vor der Katheterablation mit einer Antikoagulation begonnen werden. Die Antikoagulation sollte zudem für den Eingriff nicht unterbrochen werden. Postinterventionell sollte sie unverändert unabhängig vom CHA_2_DS_2_-VA-Score für mindestens 8 Wochen fortgesetzt werden und sich anschließend am thromboembolischen Risiko und nicht am Erfolg der Ablation orientieren. Hierzu sind in den kommenden Jahren evtl. Veränderungen zu erwarten, da möglicherweise zukünftig die Vorhofflimmerlast ein sinnvollerer Parameter für die Beurteilung einer Antikoagulationsindikation ist.

### Therapiebeurteilung und dynamische Neubewertung – wichtig, aber nicht richtig neu

Die neue Leitlinie betont, dass das Management eines Patienten mit Vorhofflimmern eine kontinuierliche Neubewertung der Behandlungsstrategie, möglicher Risikofaktoren und des Behandlungserfolgs erfordert. Es sollte eine adaptive Strategie verfolgt werden, um Veränderungen bei Risikofaktoren und Begleiterkrankungen frühzeitig zu erkennen. Gleichzeitig sollen Patienten in die Lage versetzt werden, ihre Erkrankung teilweise selbst zu managen und einzuschätzen, um unnötige Folgeuntersuchungen zu vermeiden.

## Fazit

Die aktuelle 2024 ESC-Leitlinie zum Management von Patienten mit Vorhofflimmern strukturiert mit dem zentralen Akronym AF-CARE die Vorhofflimmertherapie neu und priorisiert das Management von Komorbiditäten und Risikofaktoren. Zur Evaluation des thromboembolischen Risikos empfiehlt die Leitlinie den CHA_2_DS_2_-VA-Score zur geschlechtsunabhängigen Risikostratifikation. Eine Antikoagulation ist ab einem CHA_2_DS_2_-VA-Score von einem Punkt mit einer Klasse-IIa-Indikation, ab 2 Punkten mit einer Klasse-I-Indikation empfohlen. Bei der Wahl des Antikoagulans werden DOAC gegenüber Vitamin-K-Antagonisten empfohlen (Ausnahme: mechanische Klappen und Mitralstenose). Für subklinisches *Device-detektiertes* Vorhofflimmern wird eine zurückhaltende Empfehlung zur Antikoagulation ausgesprochen (Klasse-IIb-Indikation).

Im Laufe der letzten Dekade zeigen Daten eine fortschreitende Aufwertung der Rhythmus- gegenüber der Frequenzkontrolle. Dies spiegelt sich auch in der neuen Leitlinie wider: Eine Rhythmuskontrolle sollte bei allen geeigneten Patienten in Betracht gezogen bzw. geprüft werden. Die Katheterablation hat eine Aufwertung erfahren und wird erstmals als Erstlinientherapie für paroxysmales Vorhofflimmern im Rahmen eines *Shared-decision-Prozesses* empfohlen (Klasse-I-Indikation). Für neu diagnostiziertes Vorhofflimmern wird erstmals eine Rhythmuskontrolle zur Reduktion der kardiovaskulären Mortalität oder Hospitalisierungen (Klasse IIa) empfohlen. Bei Ineffektivität einer antiarrhythmischen Pharmakotherapie besteht zudem unverändert bei persistierendem Vorhofflimmern eine Klasse-I-Empfehlung zur Ablation.

Insgesamt setzt die Leitlinie die Entwicklung der Vorversionen zu einem ganzheitlichen, patientenzentrierten multidisziplinären Management von Vorhofflimmern fort. Sie setzt in der Betonung der Bedeutung von Begleiterkrankungen einen etwas anderen Schwerpunkt, weicht aber vom Grundpfeiler des Managements mit dem Schutz vor thromboembolischen Ereignissen nicht ab. Die Frage nach Frequenz- versus Rhythmuskontrolle verlagert sich zunehmend in Richtung einer frühzeitigen Rhythmuskontrolle, um u. a. die Progression von Vorhofflimmern aufzuhalten bzw. zu verlangsamen. Es bleibt abzuwarten, wie dies in europäischen Gesundheitssystemen mit allgemeiner Ressourcenverknappung umgesetzt werden kann.
